# Metabolic responses to the acute ingestion of two commercially available carbonated beverages: A pilot study

**DOI:** 10.1186/1550-2783-4-7

**Published:** 2007-09-14

**Authors:** Ron W Mendel, Jennifer E Hofheins

**Affiliations:** 1 Ohio Research Group – Division of Exercise Science and Sports Nutrition, Wadsworth Medical Center, Wadsworth, Ohio, 44281, USA; 2 Department of Human Performance & Sport Management, Mount Union College, Alliance, Ohio, 44601, USA

## Abstract

**Background:**

The purpose of this placebo-controlled, double-blind cross-over study was to compare the effects of two commercially available soft drinks on metabolic rate.

**Methods:**

After giving informed consent, twenty healthy men and women were randomly assigned to ingest 12 ounces of Celsius™ and, on a separate day, 12 ounces of Diet Coke®. All subjects completed both trials using a randomized, counterbalanced design. Metabolic rate (via indirect calorimetry) and substrate oxidation (via respiratory exchange ratio) were measured at baseline (pre-ingestion) and at the end of each hour for 3 hours post-ingestion.

**Results:**

Two-way ANOVA revealed a significant interaction (p < 0.001) between trials in metabolic rate. Scheffe post-hoc testing indicated that metabolic rate increased by 13.8% (+ 0.6 L/min, p < 0.001) 1 hr post, 14.4% (+0.63 L/min, p < 0.001) 2 hr post, and 8.5% (+0.37 L/min, p < 0.004) 3 hr post Celsius™ ingestion. In contrast, small (~4–6%) but statistically insignificant increases in metabolic rate were noted following Diet Coke^® ^ingestion. No differences in respiratory exchange ratio were noted between trials.

**Conclusion:**

These preliminary findings indicate Celsius™ has thermogenic properties when ingested acutely. The effects of repeated, chronic ingestion of Celsius™ on body composition are unknown at this time.

## Background

Over the past few decades, rates of obesity in the United States and many industrialized countries have risen at alarming rates [1]. This is disconcerting given the vast measures that have been taken to counter this effect. For example, the government's Food Guide Pyramid has been overhauled [2]; companies have introduced and/or reformulated their food products to promote lower carbohydrate or lower fat alternatives; schools have begun to ban junk foods from their vending machines; and several professional organizations have published guidelines regarding physical activity levels to promote weight loss [3-6]. Despite these efforts, Americans continue to gain weight [1].

Some experts have linked the increase in obesity to the consumption of sugar-containing soft drinks [7,8]. While it is well-known caffeine has a dose-dependent effect on metabolic rate [9,10], the effect of repeated (daily) use of caffeinated beverages (soft drinks, coffee, etc) on obesity has not been well characterized. Recently, several companies have released soft drink alternatives that claim to have thermogenic (calorie burning) effects above and beyond those of normal caffeinated beverages. One such product, Celsius™ is marketed as a replacement to high calorie, high sugar soft drinks, coffees, and energy drinks. Celsius™ contains 200 mg of caffeine per 12 oz, along with a proprietary blend of vitamins and phytochemicals. Many of these ingredients, particularly caffeine, guarana extract, and green tea extract can be found in capsule form as dietary supplements and have been previously shown to cause acute increases in metabolic rate [11-15]. For example, Rudelle et al. recently reported that consumption of a beverage containing mainly green tea catechins (700 mg), caffeine (100 mg), and calcium (211 mg), when consumed three times per day for three days, increased 24-hour energy expenditure (EE) by 4.6% in healthy male and female subjects [16]. Although the increase was modest, the researchers concluded that "such a beverage may provide benefits for weight control", at least when consumed as part of a prudent diet and regular physical activity.

This "proof-of-concept" study sought to determine the metabolic effects of a similar type of beverage. We surmised that determining how much of an increase in metabolic rate Celsius™ ingestion provided relative to its caloric content (in this case, ~5 Calories per 12 oz) was an obligatory step in assessing its effects on human physiology and its potential as a weight loss aid. Thus, the purpose of this study was to measure acute changes in metabolic rate after the consumption of 12 oz Celsius™, and on a separate day, 12 oz of a popular caffeine-containing soft drink (Diet Coke *®*).

## Methods

### Experimental approach

After giving informed consent and being cleared for participation by having normal blood work (assessed via comprehensive metabolic panel and lipid panel) and satisfactorily completing a health history questionnaire, twenty healthy men and women were randomly assigned to ingest 12 ounces of Celsius™ (cola) and, on a separate day, 12 ounces of Diet Coke^®^. Beverages were flavor matched and provided in unmarked bottles by EliteFx Technologies (Boynton Beach, FL). All subjects served as their own control and completed both trials using a randomized, counterbalanced design. Metabolic rate and substrate oxidation were measured via indirect calorimetry at baseline (pre-ingestion) and for 10 minutes at the end of each hour for 3 hours post-ingestion.

### Subjects

Subjects were recruited from a small suburban community in northeastern Ohio by word of mouth and posted announcements. Twenty apparently healthy subjects, men (n = 10) and women (n = 10), agreed to participate in the study (see Table 1). All subjects drank carbonated beverages (1–2, 12 oz cans) on a daily basis. Subjects were excluded from the study if they were smokers, had any known metabolic disorder, or were currently taking any medications that would impact metabolic rate. Prior to obtaining written informed consent from each subject, an institutional review board (IntegReview, Inc, Austin, TX) approved the experimental protocol. All procedures in the study were in accord with ethical standards set forth in the Helsinki Declaration of 1975, as revised in 1983.

**Table 1:** Subject Characteristics (mean ± SD)

**Gender**	**N**	**Ht (cm)**	**Wt (kg)**	**Age (yr)**	**RMR (ml/kg/min)**
Males	10	178.4 ± 4.2	83.0 ± 10.3	29.8 ± 9.2	4.13 ± 0.72
Females	10	167.7 ± 7.2	69.0 ± 11.1	30.6 ± 7.0	3.90 ± 0.43

### Metabolic rate

All subjects completed both trials in a randomized, counterbalanced order at the same time of day (0600–0800). Prior to consumption of the experimental beverages, each subject's baseline resting metabolic rate (RMR) was assessed via indirect calorimetry (TrueMax 2400, ParvoMedics, Sandy, UT) for 15 minutes. Per Compher et al., the first 5 minutes were discarded for RMR calculations [17]. This system uses paramagnetic and infrared oxygen and carbon dioxide analyzers, respectively, each with an accuracy of 0.1% and a Rudolph screen pneumotach with an accuracy of +/- 2%. Prior to each testing session, volume and gas calibrations were performed via guidelines from the manufacturer. In addition, to subjects were fitted with a standard mouthpiece, nose clip, and headgear and allowed to become familiar and comfortable with the breathing apparatus prior to collecting any expired gases. RMR was measured in the seated position after an overnight fast and at least 24 hours following intense physical activity. Diet was reproduced using 24-hr diet records recorded prior to the first trial. Immediately following the baseline RMR measurement, each subject consumed 12 oz. of either refrigerated Celsius™ or refrigerated Diet Coke^® ^within a 15 minute time period. Once the entire beverage was consumed, the subject waited for 30 minutes and another RMR measurement was made (RMR 1). This continued each hour for the subsequent two hours (RMR 2 and RMR 3, respectively). During each RMR measurement, simultaneous measurements of substrate oxidation were made via respiratory exchange ratio (RER).

### Statistical analyses

Statistical analyses were conducted using Statistica version 7.1 (Stat Soft Inc., Tulsa, OK). After confirming no three-way interactions (Beverage × Sex × Time), separate 2 (Beverage) × 3 (Time) ANOVA with repeated measures on both factors were used to analyze between group differences in RMR and RER. If a significant interaction was observed, Scheffe post-hoc testing was used to determine pairwise differences between means. Differences were considered statistically significant when the probability of type I error was less than or equal to 0.05 (P ≤ 0.05).

## Results

### Resting metabolic rate

**Figure 1:**
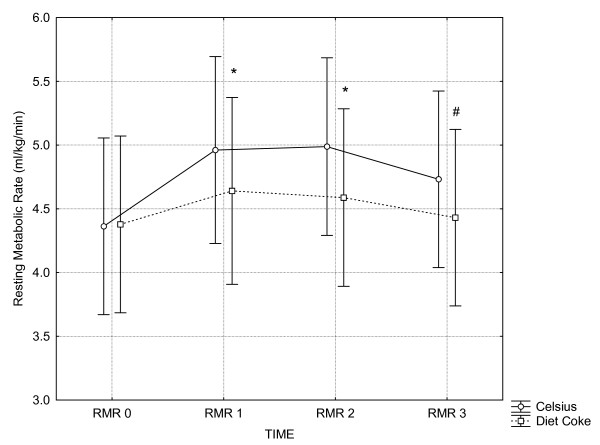
**Resting Metabolic Rate**. Vertical bars denote 0.95 confidence intervals.* p < 0.001 compared to corresponding baseline (RMR 0) value. # p < 0.004 compared to corresponding baseline (RMR 0) value.

Two-way ANOVA revealed a significant interaction (p < 0.001) between trials in metabolic rate. Scheffe post-hoc testing indicated that during the Celsius™ trial, each timepoint was significantly greater than its corresponding baseline value. Specifically, metabolic rate increased by 13.8% (+ 0.6 L/min, p < 0.001) 1 hr post, 14.4% (+0.63 L/min, p < 0.001) 2 hr post, and 8.5% (+0.37 L/min, p < 0.004) 3 hr post Celsius™ ingestion (see Figure 1). In contrast, small (~4–6%) but statistically non-significant increases in metabolic rate were noted following Diet Coke^® ^ingestion. No adverse events were reported during the study.

### Respiratory exchange ratio

**Figure 2:**
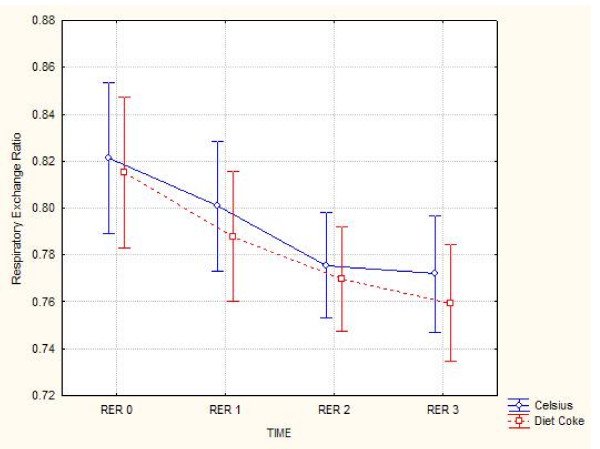
**Respiratory Exchange Ratio**. Vertical bars denote 0.95 confidence intervals. Although a time effect was noted, no significant differences were observed between trials (p < 0.90).

No differences (ANOVA interaction, p < 0.90) in respiratory exchange ratio were noted between trials (see Figure 2).

## Discussion

As a first step in a series of studies designed to assess the effects of Celsius™ when consumed as part of a daily diet, the results of this pilot study indicate that acute ingestion of Celsius™ has thermogenic properties in healthy men and women. Since RER values were not different between trials, these data also indicate that both beverages had similar effects on substrate oxidation. Although no adverse events were reported during either trial (assessed via questionnaires), it is important to note that Celsius™ contains 200 mg of caffeine per 12 oz, an amount which has been shown to transiently increase blood pressure in some normotensive subjects [18,19]. Indeed, Berube-Parent et al. reported increases of 7 and 5 mm Hg for systolic and diastolic blood pressures, respectively, in subjects who consumed 600 mg of caffeine per day, given as 3 × 200 mg throughout the day [20]. Thus, future studies should measure blood pressure responses to ascertain the potential effects of Celsius™ on systemic hemodynamics, particularly in subjects with preexisting hypertension.

Although Celsius™ contains a proprietary mixture of several physiologically active ingredients (see Table 2), it is likely that three of them, caffeine, green tea, and perhaps guarana extract are largely responsible for the observed thermogenic effects (i.e., a 12.2% increase in RMR over 3-hr post ingestion). For example, it is well known that caffeine has dose-dependent effects on metabolic rate [10,13,21,22] and that guarana extract contains a number of central nervous system stimulants with thermogenic properties (i.e., caffeine, theobromine, and theophylline). Green tea extract is also a particularly promising ingredient for enhancing thermogenesis and fat oxidation, particularly when combined with caffeine [11,12,20,23,24], however it should be noted that the amount of green tea necessary to promote these effects in humans is approximately 600 mg of total catechins per day or 300 mg per day of epigallocatechin gallate (EGCG), the active ingredient in green tea [24-29]. Unfortunately, the exact amount of catechins and/or EGCG contained in Celsius™ is not able to be determined from its Supplement Facts panel. In contrast, the only "active" ingredient in Diet Coke^® ^relative to thermogenesis is caffeine (45 mg per 12 oz).

**Table 2:** Composition of Celsius™

Serving Size 12fl. Oz		
Servings per Container 1		
Amount per serving		%DV†

Calories	5	
Vitamin C (asorbic acid)	60 mg	100%
Riboflavin	1.7 mg	100%
Niacin (as niacinamide)	20 mg	100%
Vitamin B^ (as pyridoxine hydrochloride)	2 mg	100%
Vitamin B12 (as cynocobalamin)	6 mcg	100%
Biotin	300 mcg	100%
Pantothenic Acid (as calcium d-pantothenate)	10 mg	100%
Calcium (as calcium carbonate)	50 mg	5%
Chromium (chelate)	50 mcg	41%
Sodium	6 mg	< 1%

Thermogenic Proprietary Blend	1.8 g (1,810 mg)	
Taurine		**
Guarana extract (seed)		**
Green tea leaf extract (leaf) standardized to 10% EGCG)		**
Caffeine (as caffeine anhydrous)		**
Glucuronolactone		**
Ginger extract (root)		**
Other ingredients: Carbonated filtered water, natural flavor, sucralose, artificial colors		

† Percentage Daily Values are based on a 2,000 calorie diet.

** Daily Value (DV) not established.

Rudelle et al. recently reported a 4.6% increase in 24-hour energy expenditure after subjects consumed a supplement beverage containing mainly caffeine (100 mg), green tea (700 mg [standardized to 94 mg EGCG]), and calcium (211 mg) three times per day for three days [16]. In agreement with our study, the energy contributions of carbohydrate, fat, and protein were not altered by the beverage, but the total daily energy expenditure was significantly increased by the beverage. Blood pressure, heart rate, and urinary catecholamines were also unaltered. These data also concur with those of Dulloo et al., who reported a 4% increase in 24-hour energy expenditure after the consumption of a similar mixture of caffeine and green tea (standardized for EGCG content) [12]. Although we can not exclude the possibility that other ingredients within the Celsius™ formula have supportive physiological roles, the literature to date appear to buttress the position that caffeine and green tea are the principal active components in Celsius™. For example, taurine is a conditionally essential amino acid involved in bile acid conjugation, osmoregulation, detoxification, cell membrane stabilization, and modulation of cellular calcium flux and neuronal excitability [26]. Although clinically useful in the treatment of cardiovascular disease, hypercholesterolemia, seizure disorders, ocular disorders, diabetes, hepatic disorders, cystic fibrosis, and alcoholism, we are unaware of any studies demonstrating a direct effect of taurine on RMR. Similarly, glucuronolactone is a glucose metabolite produced in the liver that is purported to improve concentration and memory [27]. Finally, ginger extract has been reported to have anti-nausea effects and may also have anti-inflammatory properties [28]. As with taurine, the effects of glucuronolactone and ginger extract on RMR are unknown. Future studies should determine the effects of these popular ingredients on energy metabolism so that their contribution (if any) to the observed results can be delineated.

## Conclusion

These preliminary findings indicate that acute consumption of Celsius™ stimulates thermogenesis to a greater extent than Diet Coke^®^, at least over three hours post-ingestion in healthy men and women. These findings are not surprising considering that Celsius™ contains 4.4 times more caffeine than Diet Coke^® ^per 12 oz. It is tempting to speculate that daily consumption of Celsius™ might improve body composition over several weeks of continuous use, particularly when some epidemiologists have estimated that a caloric deficit of as little as 100 kcal/d may be sufficient to prevent weight gain in the majority of Americans [29]. However, this assumes that the effects we observed on metabolic rate are sustained and not attenuated during chronic use. It is our understanding that these types of studies are currently being planned by other research groups, therefore the long-term safety and efficacy of Celsius™ remains to be demonstrated.

## Competing interests

The presentation of results of this study does not constitute endorsement by any of the researchers or their affiliations. The authors declare that they have no competing interests.

## Authors' contributions

RWM participated in the design of the study, assisted in data collection, performed the statistical analysis, interpretation of data, and drafted the manuscript. JEH participated in its design, coordinated and collected data and helped to draft the manuscript. All authors read and approved the final manuscript.
